# Novel 3D micro- and nanofabrication method using thermally activated selective topography equilibration (TASTE) of polymers

**DOI:** 10.1186/s40580-014-0007-5

**Published:** 2014-02-28

**Authors:** Arne Schleunitz, Vitaliy A Guzenko, Martin Messerschmidt, Hakan Atasoy, Robert Kirchner, Helmut Schift

**Affiliations:** 1 Paul Scherrer Institut, Laboratory for Micro- and Nanotechnology, 5232 Villigen, PSI Switzerland; 2 Micro Resist Technology GmbH, Koepenicker Str. 325, 12555 Berlin, Germany

**Keywords:** Thermoplastic polymer, Thermal reflow, Three-dimensional, Surface topography, Electron beam lithography, Nanoimprint lithography, Nanofabrication

## Abstract

Micro- and nanostructures with three-dimensional (3D) shapes are needed for a variety of applications in optics and fluidics where structures with both smooth and sharp features enhance the performance and functionality. We present a novel method for the generation of true 3D surfaces based on thermally activated selective topography equilibration (TASTE). This technique allows generating almost arbitrary sloped, convex and concave profiles in the same polymer film with dimensions in micro- and nanometer scale. We describe its principal mechanism exemplified by pre-patterned poly (methyl methacrylate) resist which is exposed to high energy electrons prior to a thermal annealing step enabling the selective transformation of stepped contours into smooth surfaces. From this we conclude, that TASTE not only offers an enormous degree of freedom for future process variations, but also will advance the patterning capabilities of current standard 3D micro- and nanofabrication methods.

## Background

Current applications in micro- and nanotechnology mainly use binary (i.e. two-level) surface topographies provided by state-of-the-art planar fabrication processes. However, the increasing technical requirements for new or enhanced device functionality with sophisticated 3D topographies have to be met. This includes regular sub-wavelength blazed gratings and microprisms for out-coupling of light into planar waveguides for optical application [1,2], as well as shallow tapered inlets bridging micro- to nanofluidics for life-sciences [3,4]. Currently, 3D topographies are often simply approximated by steps with adapted widths and heights which resemble provisional and quasi-3D contours. In this case, viable pattern fidelity is connected to high process complexity since non-continuous multilevel features need multiple lithographic and etching [5] or electroplating steps [6]. Alternative approaches for continuous contours using anisotropic etching of crystalline silicon in alkaline solutions are restricted to specific geometries [7,8]. Thus novel process strategies are required in order to overcome the limitations of generic micro- and nanofabrication techniques.

By implementing the TASTE method (i.e. thermally activated selective topography equilibration), we demonstrate the possibility to generate truly 3D shaped topographies with decreased technical effort while the geometrical diversity is enhanced at the same time. TASTE is a further development and generalization of an effect observed during selective thermal reflow of grayscale structures, presented in [9]. In this original process grayscale lithography and thermal reflow were combined, i.e. a positive resist was locally exposed with a modulated dose of high energy (100 keV) electrons, which enabled to create a height profile with distinct steps after development in a suitable solvent. The stepped structures could be transferred into a linear slope by selective reflow by heating to a moderate temperature. This was possible because the exposure led to the simultaneous local modification of both etch rate and glass transition temperature T_g_ over the entire film thickness. This led to distinct heights of remaining resist during development and selectivity during reflow, both between exposed structures which could be modified and unexposed structures which remained unaffected. Apart from this pronounced selectivity, the basic principles of the selective reflow are:

- the high thermo-mechanical selectivity between exposed and unexposed resist
- a local reflow and redistribution of polymer of only a few steps
- the definition of the final shape due to volume conservation and pinning on the interface to the unexposed structures and to the cleared substrate.

**Figure 1:**
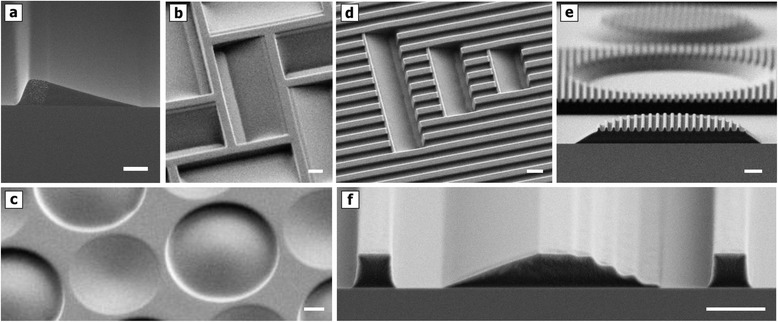
**Compilation of exemplary 3D contours in a thin PMMA film enabled by the TASTE method.** The SEM micrographs (angled views and cross sections) depict refined PMMA topographies after exposure of pre-patterned PMMA to high energy electrons and subsequent thermal annealing. Achievable contours comprise **(a-c)** binary, stepped, sloped, convex and concave structures, as well as **(d-f)** hybrid structures with specific pattern combinations. (scale bars: 1 μm).

As can be seen in the compilation of exemplary 3D structures illustrated in Figure 1, the variety of achievable structures comprises the creation of stepped, sloped, concave and convex contours, with a range of slope angles and curvatures, as well as sizes, orientations and density variations in the very same polymer surface. In all cases TASTE was applied to a 1 μm thick poly(methyl methacrylate) resist (PMMA) on a silicon substrate resulting into diverse 3D topographies exhibiting exceptionally smooth contours, enabled by the selective transformation of stepped surfaces into new continuous topographies during a single thermal annealing step. Up to now, slope angles of up to 45° in 2 μm thick resists with optically smooth surfaces with a few nm roughness were achieved [9–11].

### Background: Fundamental working principle

**Figure 2:**
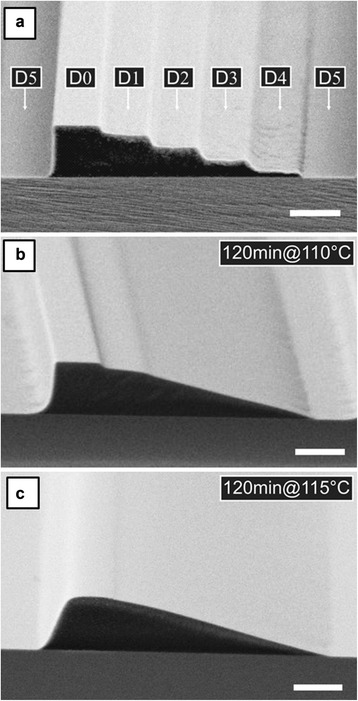
**SEM micrographs of the selective thermal reflow of grayscale structures in PMMA: a) The stepped contour with five discrete levels (width: 1 μm; step height: ~ 200 nm) was generated in a 1 μm thick resist by local exposure to high energy (100 keV) electrons with doses D1 to D5 and wet development in pure MIBK.** The annealing step was performed for 120 min at 110°C **(b)** and 115°C **(c)**. While for 110°C the step nearest to the unexposed PMMA is still visible, for 120°C it is smoothened out. (scale bars: 1 μm).

As fundamental aspect of TASTE, we have previously identified the local variations of viscosity in the electron-beam exposed PMMA resist during the heating step, which originates from the dose dependent reduction in molecular weight M_w_ (here weight-averaged molecular weight) and glass transition temperature T_g_ of the polymer. A typical asymmetric 5-level grayscale PMMA structure is depicted in Figure 2 to which locally five different doses (D1 - D5) were applied according to a specific lateral design, prior to development in pure methyl-isobutyl-ketone (MIBK). Here, D0 represents an unexposed area, where the initial resist thickness is preserved, while D5 corresponds to the dose required to completely clear the resist upon development, i.e. dose-to-clear. Since for a stepped structure the highest step (D1) will have M_w_ nearest to the original M_w_ (D0 = 0), this results also in a reduced ability to flow. This can be reduced by using a high enough reflow temperature near to the T_g_ of the unexposed ridge, leading to a softening and thus slight inclination. A simplification can therefore be achieved if an already produced slope structure with a homogeneous M_w_ is generated, e.g. by using an imprinted thermoplastic resist structure such as PMMA which is flood exposed with a dose D > D1 over all steps. Using this, all steps can be exposed to generate low M_w_, which enables a complete reflow and an equal smoothening, particularly also the uppermost step of the 5-level pattern with the lowest reduction in M_w_. Furthermore it enables to do the reflow at low temperature, at which the non-exposed ridge with a high M_w_ is less affected by the reflow step. Aiming for a versatile and robust process, our investigations were focused on defining thresholds which enhance the selectivity in reflow behavior for a range of surface topographies, i.e. a selective transformation of structures into defined contours while others remain unaltered. For this we have analyzed the specific correlation between M_w_ and resulting T_g_ of the exemplary PMMA resist used in this work.

**Figure 3:**
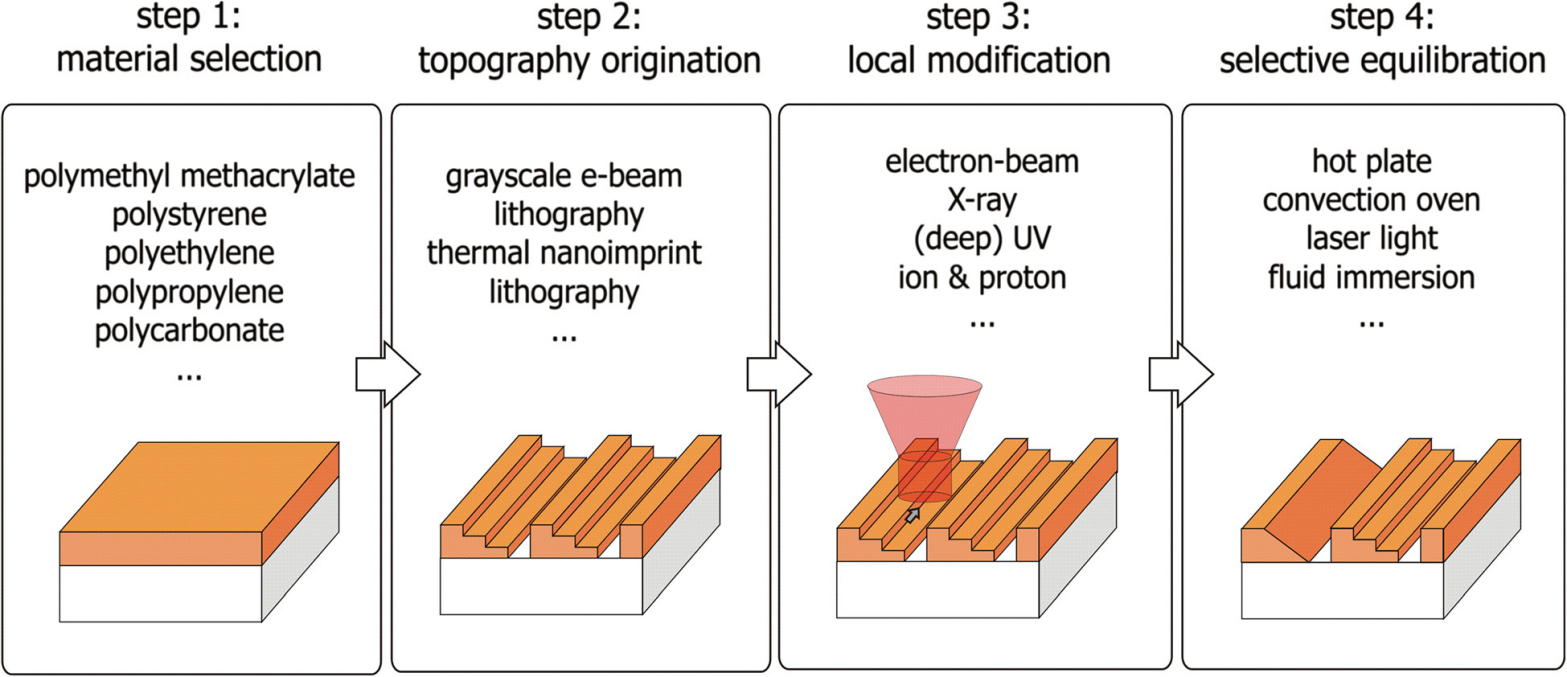
**Schematic flow sequence of the general TASTE method for the generation of asymmetric 3D blazed grating pattern with linear sloped and stepped contours: pre-treatment of selected polymer films, generation of a specific (stepped) topography, local modification of the polymer’s thermo-mechanical properties and thermal treatment for enabling a selective topography equilibration.**

Before a detailed technical discussion is given further below, the basic process sequence of the generalized TASTE method is illustrated in Figure 3 for the generation of asymmetric 3D grating elements with linear sloped and stepped contours. Here, a thermoplastic polymer material is deposited on a solid substrate (*step 1*: material selection) and patterned with a specific stepped topography, e.g. using standard lithographic methods such as grayscale electron-beam lithography [12–15] (*step 2*: topography origination). Next, the polymer material is precisely modified over selected steps, e.g. upon irradiation to high energy electrons using defined doses (*step 3*: local modification), if not already done by grayscale lithography. This way, the thermo-mechanical properties of the polymer are locally adjusted, allowing an optimized transformation of the exposed stepped contours into smooth slopes, if the polymer is subsequently heated to an appropriate temperature (*step 4*: selective equilibration). Although the annealing step is performed to the entire film-coated substrate, only the locally exposed polymer structures are softened which allows surface tension driven topography equilibration to selectively convert steps into smooth slopes. Since the original shape of unexposed areas stays unaltered upon thermal treatment, the generation of entirely novel topographies is enabled which includes vertical, stepped and slopes contours in close vicinity to each other (see Figure 1f). This makes TASTE a unique method since it is different to traditional thermal reflow at higher temperatures which aims for the collapse into spherical or cylindrical shapes or decay of all structures [16–18].

The combination of material and lithography used in this contribution, i.e. PMMA and electron beam lithography (EBL), is only one specific example of a TASTE method which allows a precise control of resolution, dose and alignment in a well-defined material. The schematic in Figure 3 also specifies possible alternative materials and sub-processes indicating the high degree of freedom for alternative technical implementations of TASTE. This contribution has two aims: To review the achievements and possibilities of TASTE and to present an analysis of the relation exposure dose to M_w_ and T_g_, which is the cornerstone of TASTE. By this we will demonstrate the width of process window possible by exposure with standard EBL. At the end we will give an insight about how shapes of TASTE structures could be predicted by simulation using a soapfilm-based model.

## Methods

The PMMA for spincoating is an experimental sample provided by micro resist technology GmbH (Germany), with a specified weight averaged molecular weight M_w_ of 120 k (kg mol^-1^). As it will be shown below, the actual M_w_ is much lower than 120 k, and for comparison, it is much more convenient to use M_n_ (number-averaged molecular weight) instead of M_w_. In comparison to high M_w_ PMMA generally used for EBL, this M_w_ was chosen to be low enough to enable a prepatterning of resist using nanoimprint lithography (NIL) (see Figure 1e) [19,20]. This is possible since the typical temperature of imprint (160-180°C) and the T_g_ of the unexposed resist (122°C) are different enough to enable a selective reflow of exposed structures without affecting imprinted structures [21]. Furthermore the M_w_ was found high enough to enable a selective reflow of exposed structures with M_w_ below the T_g_-critical threshold. The raw (i.e. unexposed) polymer material was predominantly syndiotactic PMMA (7% isotactic, 54% syndiotactiv, 39% atactic; determined by ^1^H-NMR spectroscopy). It was dissolved in anisole enabling the spin-coating deposition of 1 μm thick layers on silicon substrate. For the PMMA resist being exposed to different doses, the distinct reduction of the molecular weight distributions was determined using gel permeation chromatography (GPC), and the T_g_ was analyzed using differential scanning calorimetry (DSC), by removing flakes of exposed PMMA films from the substrate. EBL exposure was performed in a Vistec EBPG 5000 Plus ES. The corresponding contrast curve can be found elsewhere [11]. After exposure, a stepped contour with five discrete levels (step width: 1 μm) was generated in a 1 μm PMMA resist after wet development in pure methyl-isobutyl-ketone (MIBK) for 30 s at 20°C. Because the aim was to create a linear slope between the top of the ridge and the substrate as two pinning points, in between a stepped slope with four intermediate steps needed to be placed. This was possible by choosing the step heights different from the average step height of ~ 200 nm, with a larger step in the center of the slope. This was done according to volume conservation over the entire slope as well as within the vicinity of each step. The annealing step was performed for 120 min at different temperatures near the T_g_ of the unexposed resist, on a hot plate in ambient condition. Analysis was done via scanning electron microscopy (SEM).

## Results and discussion

### Polymer analysis

**Figure 4:**
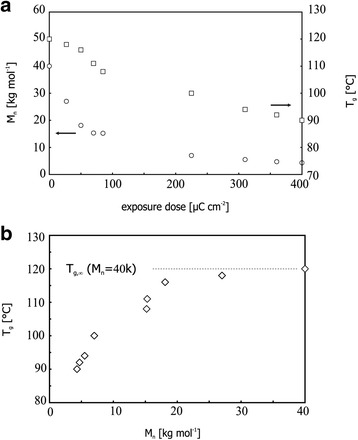
**Dependencies of the exposure dose, the (number averaged) molecular weight M**_**n**_ **and the glass transition temperature T**_**g**_ **for electron-beam exposed PMMA films which is used in this contribution by selective topography equilibration (i.e. TASTE).** When the material properties of PMMA resist are modified by exposure to high energy (100 keV) electrons **(a)**, the resulting correlation of T_g_ over M_n_ shows a smooth transition **(b)**. By creating a wide range of T_g_, the selective shape transformation of the exposed (and thus T_g_ lowered) resist is accomplished, when the reflow temperature is adequately chosen, e.g. at least 10 K higher than the highest T_g_ present in the PMMA contour.

In order to assess the optimum reflow conditions such as the temperature, the GPC measured M_n_ data were correlated to specific T_g_ by using DSC. The results are graphically summarized in Figure 4a and quantified by the averaged masses derived from the GPC data presented in Table 1: the exposure induced decay of M_n_ implicates a significant reduction of the T_g_, when the dose is 225 μC cm^-2^ or higher. This results in a reduction of the T_g_ of unexposed PMMA (initially at T_g,∞_ = 122°C) of more than 20 K to temperatures in the range of 90 – 100°C. The grayscale steps can therefore be selectively transformed into continuous slopes, while the non-exposed ridge is less affected by the reflow step. It is particularly interesting that for PMMA exposed to doses 225 μC cm^-2^ or higher, the M_n_ of initially 40 kg mol^-1^ is reduced to 7 kg mol^-1^ and lower. This leads to altered thermo-mechanical properties of the exposed PMMA since the material cohesion and thus viscosity is changed in particular, when the polymer chain length is below a critical [22] M_n_ of about 10 kg mol^-1^. Below this threshold, the chain entanglement is substantially reduced and thus thermally induced chain motions are less retarded, i.e. topological restriction by neighboring chains becomes less important. When the polymer is heated, the induced thermal energy is consumed by activating cooperative translation movements of the polymer molecules. For a decreased M_n_, a polymer chain motion is enhanced more easily due to the increased free volume in the polymer resulting from the increased presence of chain ends. This results in a softening of exposed polymer material already at reduced temperatures which in turn corresponds to a decreased T_g_ [23]. The variation of a few degrees in T_g_ results in viscosity changes of orders of magnitude [6] for a given temperature. In the ideal case, flow only occurs between neighboring exposed steps (i.e. in “near field”), which can be accomplished when using proper reflow conditions and thus maintain a sufficient material viscosity. This prevents the formation of cylindrical or spherical pattern as are known from classical reflow processes [16–18]. Consequently, PMMA features exposed to high energy electrons can be selectively reflowed at eligible temperatures while unexposed pattern stay unaltered.

**Table 1:** **Summary of the GPC and DSC measurements quantifying the dose dependencies of the thermo-mechanical properties of PMMA**

**Exp. dose [μC cm** ^**-2**^ **]**	**M**_**n**_ ** [kg mol** ^**-1**^ **]**	**M**_**w**_ **[kg mol** ^**-1**^ **]**	**PD [-]**	**T**_**g**_ ** [°C]**
*unexposed*	40.0	91.2	2.0	122
27	27.0	58.3	2.2	118
50	18.1	45.2	2.5	116
70	15.3	35.0	2.3	111
85	15.2	34.0	2.2	108
225	7.0	14.6	2.1	100
310	5.5	11.5	2.1	94
360	4.7	10.0	2.1	92
400	4.3	8.9	2.1	90

Upon exposure to high energy electrons, the number averaged molecular weight M_n_, weight averaged molecular weight M_w_, polydispersity PD (M_w_/M_n_) and glass transition temperature T_g_. is modified according to the applied doses.

The topography transformation of the exposed areas is enabled by surface tension forces which are oriented parallel to the interface (polymer/ambience) [24] and thus are imbalanced for stepped contours with kinks (protrusions) and edges (depressions). When the PMMA resist is locally softened, the unbalanced surface tension forces initiate localized material movements until the polymer surface is in a favorable energetic state (e.g. which in high temperature reflow is based on pinning and surface minimization where the surfaces are in equilibrium). For stepped 3D resist profiles with a pinning at the unexposed ridge and the cleared substrate, this state is reached for a continuous linear slope. The resulting angle of inclination is predetermined by the pinning and initial geometry. Since the reflow process is based on kinetic effects, which takes place with different progression speeds correlated to the given viscosity variation, more than a mere smoothening into linear slopes is enabled. Convex and concave pattern of distinct shapes (e.g. non-cylindrical and -spherical, which is advantageous over standard reflow) are also possible (see Figure 1c) because the material displacement during smoothening is locally confined. This allows predefining non-linear trajectories based on contour approximation and volume conservation.

### Stepped and sloped sidewalls in close vicinity

Correlating the GPC and DSC data allows drawing T_g_ directly as a function of M_n_, which reveals a rather smooth and gradual decline of T_g_ (Figure 4b). Thus, an abrupt change in thermal properties for pattern with critical M_n_ of lower than 10 kg mol^-1^, as anticipated previously [22], is not confirmed. This leads to an empirically found general definition of TASTE compatible process windows for the PMMA used in this work, which can be described as follows: an optimal reflow temperature is given at least 10 K below the polymers original T_g_, and 10 K above the highest T_g_ present in the polymer pattern, respectively. For a chosen temperature of 110°C, these prerequisites are only achieved for generating PMMA patterns with a maximum M_n_ of 5 kg mol^-1^, which according to Figure 4a corresponds to an exposure dose of 400 μC cm^-2^ or higher.

**Figure 5:**
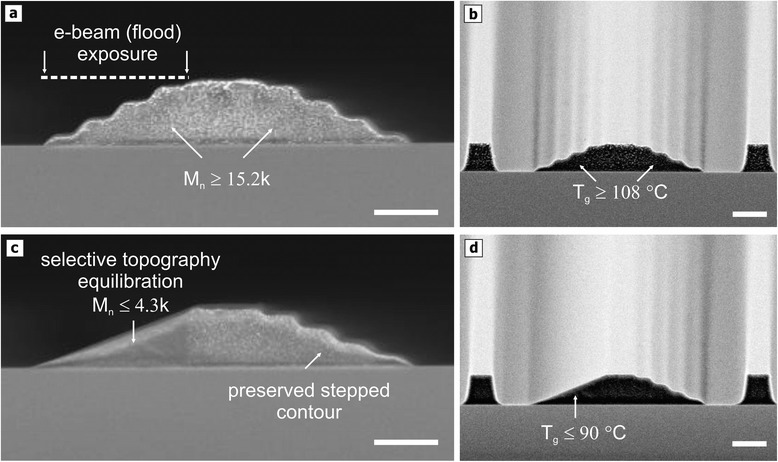
**A partially (flood) exposing a multilevel PMMA resist contour within the TASTE process enhances the available pattern variety of 3D structures.** The generation of sloped and stepped contours in very close vicinity to each other is enabled by locally exposing a stepped PMMA contours **(a-b)** with high dosage (i.e. 500 μC cm^-2^) and thus reducing the T_g_ below 92°C. Applying a reflow temperature of 110°C, exposed steps are then selectively transformed into a continuous slope while other topographies are unaltered **(c-d)**. Partial flood exposure also optimizes the overall slope accuracy since the thermal properties of the exposed areas are homogenized (scale bars: 1 μm).

This way, stepped and sloped topographies can be combined in close vicinity, as demonstrated in Figure 5. Here, the stepped polymer patterns (either fabricated by grayscale EBL or 3D NIL) prior to thermal treatment, i.e. the stepped patterns, are exposed by a well aligned local flood exposure, enables the appropriate reduction of M_n_ into a favorable regime for reflow with sufficiently reduced T_g_. In contrast to previous works by the authors, where grayscale EBL was used for topography origination which led to a rather unspecific modification of the material properties [25], this defines effective M_n_ values in the resist not correlated to distinct level heights. This way, typical reflow defects at upper most level height reported before are overcome and the overall slope characteristics are optimized (see also Figure 2).

The broad T_g_ distribution depicted in Figure 4a and 4b achieved by the electron-beam exposure also allows to further advance the available pattern variety, when two identical stepped resist contours are generated in PMMA resist, as shown in Figure 5, using a grayscale EBL with doses between 25 and 85 μC cm^-2^ and a respective development time of 15 min. Here, the comparatively low doses result in a moderate reduction of M_n_ >> 10 kg mol^-1^ for the stepped pattern and thus into marginal decrease of T_g_ not less than 108°C. A subsequent and locally confined flood-exposure of 500 μC cm^-2^ further reduces (and eventually homogenizes) the M_n_ range within the exposed steps below 5 kg mol^-1^, which results in a reduced T_g_ < 90°C. If a thermal treatment at a fixed temperature of 110°C for 15 min is now applied, only the exposed steps are selectively transformed into a linear slope. The non-flood exposed contours, which have still relatively high T_g_, stay unaltered upon reflow since the viscosity remains high. Future advancement can be achieved when an overlap of this flood exposure with the central non-exposed ridge ensures an optimal slope pinning to the upper level without any kink and consequently enhances the overall characteristics of 3D topographies.

### Simulation of reflow using soap film-based model

**Figure 6:**
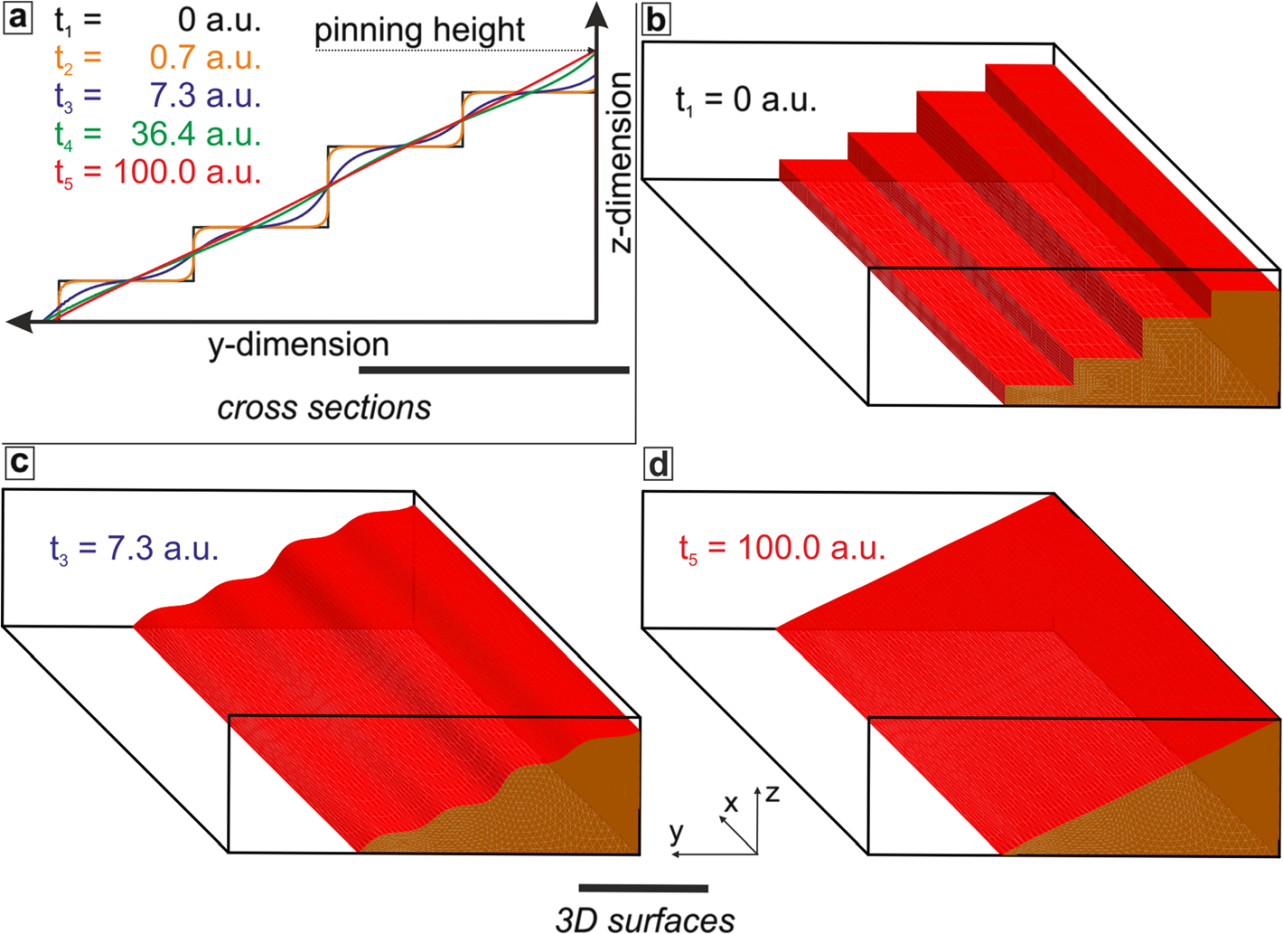
**Simulation results for the EBL flood exposure area in Figure** 5 **, using the true experimental geometry.** The simulation visualizes the time dependent multi-step evolution from the initial shape (t_1_) to the shape at the end of the reflow (t_5_) by **a** **)** cross sections and **b-d** **)** 3D surface representations. The highest step is pinned during reflow at the top of the unexposed PMMA part (scale bars: 1 μm).

For many applications not only the final shape, but also the pattern evolution from the initial into the reflow shape (Figure 2) depending on material and process parameters would be of interest. A simulation based on the time-temperature dependent, dynamic evolvement of the polymer surface during the reflow process was performed, based on open source software [26]. As an example, the case of a six level (five exposure-level) structure was simulated (see Figure 6), similar to that in the experiments presented in Figure 5, with an unexposed ridge on the right side and the cleared substrate on the left side. The major influencing parameters during reflow are i) the initial feature geometry after electron beam exposure, ii) the material properties of the reflown thermoplast, and iii) the reflow parameters itself. The influence of all these parameters on the shape evolution is implemented in a soapfilm-based model [26] via a dynamic contact angle between the polymer and the surface wetted by the polymer [27]. The discrete-time model allows determining precisely a point at which the relaxation should be stopped by reducing the sample temperature well below the glass transition to freeze a certain curvature. Using this, in the simulation presented in Figure 6, it is possible that the pinning at the lower end of the slope can be nearly preserved, while the polymer is able to rise up to the top of the unexposed ridge. Apart from pinned slopes, more complex structures can be predicted, including concave and convex shapes. Details can be found in [28].

## Conclusions

The enormous degrees of freedom offered by the TASTE method brings us to conclude that the process sequence of stepped topography origination, flood exposure and reflow in a thermoplastic polymer layer needs to be specified as a new lithographic method for 3D patterning. Compared to previous works, the increased reflow selectivity and improved pattern accuracy with less geometrical defects, by introducing a flood exposure to pre-patterned polymer contours, enabled contour transformation to be performed in a pre-determined way almost entirely given by geometry. As already indicated in Figure 3, process variants could employ other polymers (e.g. polystyrene) as *step 1*, which are also known for its nanofabrication capabilities by lithographical means [29,30], or resists suitable for NIL [21]. For topography origination of stepped structures as *step 2*, instead of patterning by grayscale EBL, replication by 3D-NIL could be employed. This allows the replication of diverse preliminary (e.g. stepped, corrugated, defective) structures originating from a range of processes which can be subsequently transformed into structures with desired shape and functionality by localized flood (over entire slopes) or selected area (up to global) exposure. In future, it might also be interesting to explore the possibilities of alternative exposure techniques as *step 3* by exposure to X-rays, ions, proton or (deep) UV-light, also well known to modify the thermo-mechanical properties of resists [31]. As *step 4*, global heating schemes may involve a hotplate and convection oven, but can also include local treatment by laser light irradiation or immersion in heated fluids. Typically, once such complex features are fabricated, it can be used as pattern origination in mass-replication techniques like NIL or other molding processes, where 3D surface reliefs are repeatedly generated by spatial material displacement [19,20]. The potential of the TASTE method towards entirely novel 3D profile variants is high and will direct towards new applications where those varieties are needed.

## Abbreviations

TASTE: Thermally activated selective topography equilibration
3D: Three-dimensional
PMMA: Poly(methyl methacrylate)
M_w_: Weight-averaged molecular weight
M_n_: Number-averaged molecular weight
T_g_: glass transition temperature
MIBK: Methyl-isobutyl-ketone
EBL: Electron-beam lithography
NIL: Nanoimprint lithography
SEM: Scanning electron microscopy
GPC: Gel permeation chromatography
DSC: Differential scanning calorimetry
